# Experimental, DFT and MD Assessments of Bark Extract of *Tamarix aphylla* as Corrosion Inhibitor for Carbon Steel Used in Desalination Plants

**DOI:** 10.3390/molecules26123679

**Published:** 2021-06-16

**Authors:** Ismat H. Ali

**Affiliations:** 1 Department of Chemistry, College of Science, King Khalid University, P.O. Box 9004, Abha 61413, Saudi Arabia; ihali@kku.edu.sa; 2 Research Centre for Advanced Materials Science (RCAMS), King Khalid University, P.O. Box 9004, Abha 61413, Saudi Arabia

**Keywords:** corrosion, inhibition, barks, *T. aphylla*, carbon steel, seawater

## Abstract

This study aimed to examine the extract of barks of *Tamarix aphylla* as a corrosion inhibitor. The methodology briefly includes plant sample collection, extraction of the corrosion inhibitor, gravimetric analysis, plotting potentiodynamic polarization plots, electrochemical impedance spectroscopic measurements, optimization of conditions, and preparation of the inhibitor products. The results show that the values of inhibition efficiency (IE%) increased as the concentrations of the inhibitor increased, with a maximum achievable inhibition efficiency of 85.0%. Potentiodynamic polarization (PP) tests revealed that the extract acts as a dual-type inhibitor. The results obtained from electrochemical impedance spectroscopy (EIS) measurements indicate an increase in polarisation resistance, confirming the inhibitive capacity of the tested inhibitor. The adsorption of the inhibitor on the steel surface follows the Langmuir adsorption isotherm model and involves competitive physio-sorption and chemisorption mechanisms. The EIS technique was utilized to investigate the effect of temperature on corrosion inhibition within the 298–328 K temperature range. Results confirm that the inhibition efficiency (IE%) of the inhibitor decreased slightly as the temperature increased. Lastly, the thermodynamic parameters for the inhibitor were calculated.

## 1. Introduction

Corrosion is a treacherous phenomenon that may demolish metals and alloys and diminish the efficiency of metallic and alloyed products and shorten their lifetime. Corrosion problems affect most of the industrial sectors in Saudi Arabia. The probable cost of corrosion based on the gross national products (GNP) of Saudi Arabia in 2011 was approximately 25 billion USD, while the corrosion cost in 2004 was about 18 billion USD. This indicates that corrosion in Saudi Arabia is a growing problem [1].

It is known that Saudi Arabia produces a vast amount of desalinated water (i.e., more than 1.5 billion cubic meters per year). The total corrosion cost in plants for seawater desalination in the Kingdom was estimated in 2007 to be 400 million SR. This cost increased in 2008 to approximately 500 million SR. These statistics indicate that the corrosion problem in seawater desalination plants in Saudi Arabia needs more attention than before.

Usually, corrosion treatment is carried out by adding synthesized organic chemicals to metallic and alloyed products. One of the best techniques used to protect metals and alloys from corrosion is using inhibitors. Organic compounds having heteroatoms, π electrons in triple and electronegative functional groups, or conjugated double bonds usually are applied to diminish the corrosion attack on metals in acidic media [2,3,4]. Generally, those compounds are adsorbed on the surface of a metal. Hence, they block the active corrosion positions. However, there are several problems related to chemical inhibitors, such as their high cost and the negative effect on the environment.

In recent years, extracts of natural substances have attracted some attention for their potential as eco-friendly, non-toxic, and biodegradable corrosion inhibitors [5,6,7,8,9,10]. These constituents can be obtained using inexpensive and straightforward techniques. It is also reported that gum acacia has efficient inhibition properties against steel corrosion [11].

The positive results obtained for natural constituents have motivated us to examine the use of the extract of barks of *T. aphylla*, abbreviated as BTA, collected from the Asser area in the south of the Kingdom of Saudi Arabia, as a corrosion inhibitor for carbon steel in a seawater medium. In addition to potentiodynamic polarization and impedance spectroscopy, the Gravimetric method was used to study corrosion inhibition behavior. Theoretical studies and scanning electron microscopy were also used to study the inhibition properties of the BTA.

## 2. Results

### 2.1. Phytochemical Screening of the Crude Extracts

Phytochemical test of barks of *T. aphylla* indicated that it contained flavonoids, phenolic compounds, glycosides, tannins, steroids, and Saponins. On the other hand, the barks tested negative for alkaloids and anthraquinones.

### 2.2. Weight Loss Measurements

Various BTA concentrations were added at 298 K to the corrosive medium in order to examine the effect of BTA concentration on the inhibition process. The corrosion rate in millimetres per year (mm/y) was determined by Equation (1) [9]:

(1) $$C.R=\frac{K\;\times \;W}{A\;\times \;t\;\times \;\rho }$$

where the constant K is equal to 8.76 × 10^4^, and w and t represent the mass loss in grams and the exposure time in hours, respectively. The inhibition efficiency ${\mathrm{IE}}_{\mathrm{WL}}\;\%$ and the surface coverage (θ) were determined by Equations (2) and (3) [9]:

(2) $${\mathrm{IE}\%}_{\mathrm{WL}}=\left[\frac{C_{R}^{o}-{\;C}_{R}}{C_{R}^{o}}\right]\times 100$$

(3) $$\;\theta =\left[\frac{C_{R}^{o}-{\;C}_{R}}{C_{R}^{o}}\right]$$

where $C_{\mathrm{RW}}$ and $C_{\mathrm{RW}}^{o}$ are the corrosion rates in the presence and absence of various BTA concentrations, respectively, and θ is the degree of surface coverage. Table 1 summarizes the obtained results. The rates of inhibition efficiency were found to increase continuously with an increase in concentration. This response supports the assumption that by increasing the concentration of the inhibitor, the adsorption of the inhibitor will gradually increase, leading to a complete blockage of corrosion of active sites except for 1-θ of the exposed surface area [11].

### 2.3. Stability of the Inhibitor

**Figure 1:**
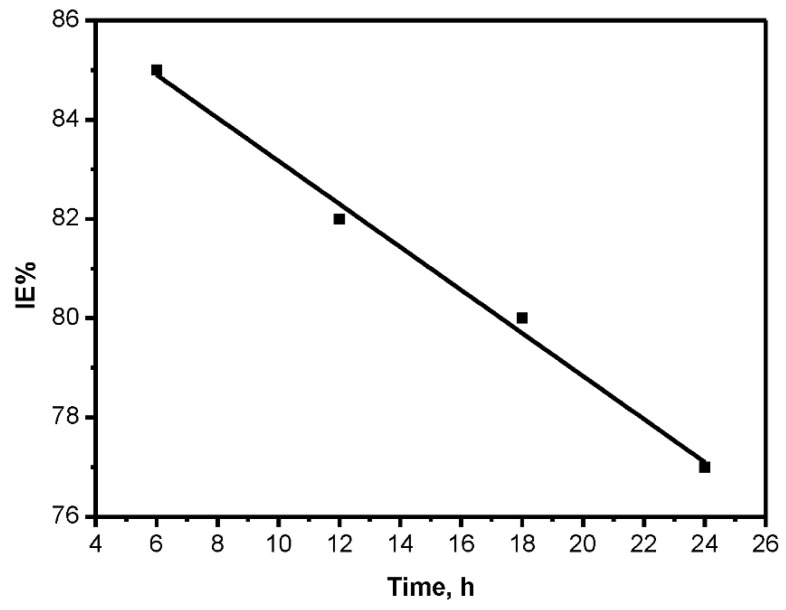
Effect of immersion time on the inhibition efficiency for carbon steel in sea water at 298 K.

To evaluate the stability of the inhibitive layer of the BTA and to conclude the time needed for the inhibitor to reach the extreme inhibition effectiveness, weight loss tests were accomplished on carbon steel samples in seawater with the optimum concentration of the inhibitors for various immersion times. The results are displayed in Figure 1. The inhibition effectiveness decreased as immersion time increased. In addition, a higher effectiveness was recorded at 1–6 h of immersion. When the immersion time was raised to 24 h, a noticeable reduction in effectiveness was observed. This behavior is due to the instability of the biodegradable nature of the plants’ extract after prolonged contact [12,13].

### 2.4. Electrochemical Measurements

#### 2.4.1. Potentiodynamic Polarization Plots

**Figure 2:**
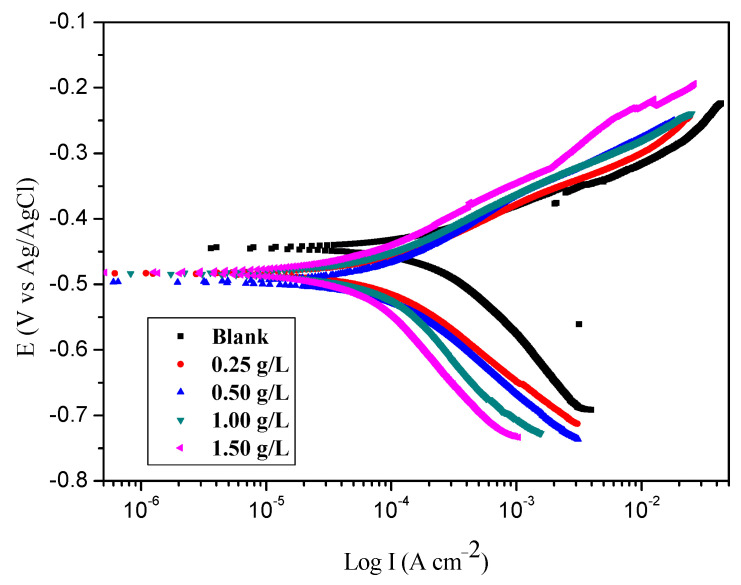
Potentiodynamic polarization plots of steel in sea water in the presence of different concentrations of BTA.

Figure 2 shows the polarization plots of numerous concentrations of the BTA. The corrosion current density (I_corr_) viz. the kinetic factors, anodic Tafel slopes, cathodic Tafel slopes (bc), and corrosion potential (E_corr_) were accomplished from these plots and are presented in Table 2. Values of inhibition efficiency (EI%) were determined using Equation (4):

(4) $$EI\%=\frac{I_{\mathrm{corr}}^{o}-{\;I}_{\mathrm{corr}}}{I_{\mathrm{corr}}^{o}}\times 100$$

where I°_corr_ and I_corr_ represent corrosion current densities of the steel samples, without and with the inhibitors, respectively.

Results in Table 2 indicate that the values of I_corr_ decreased steadily with the increase of the concentration of the inhibitor. The values of the anodic Tafel slopes b_a_ and cathodic Tafel slopes b_c_ differ slightly except at the lowest concentration, which indicates that the proton discharge reaction mechanism did not change the inhibition mechanism.

Figure 2 reveals that the BTA suppressed both anodic and cathodic currents, confirming a dual-type inhibitor. The inhibition efficiency (IE%) increased as the inhibitor concentration increased, reaching a maximum value of 84.8% at 1.50 g/L.

#### 2.4.2. Electrochemical Impedance Spectroscopy (EIS)

**Figure 3:**
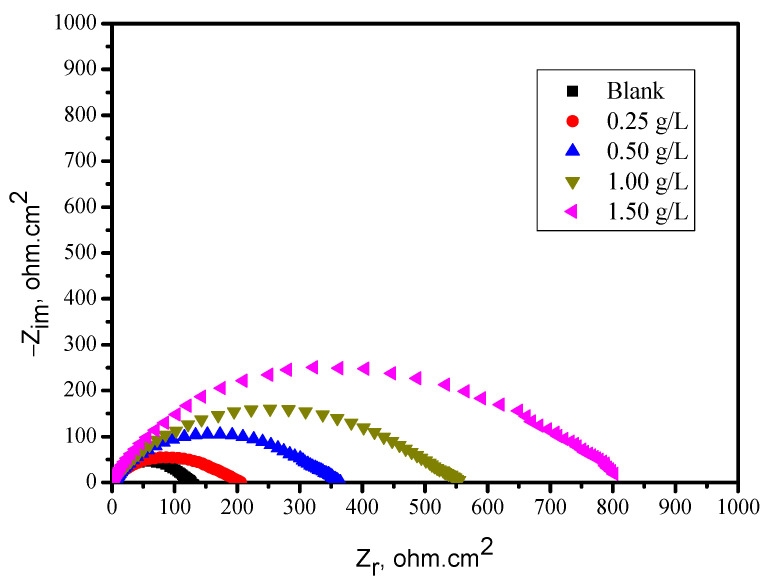
Nyquist plots for carbon steel electrode with and without the BTA.

The EIS experiments were conducted in uninhibited and inhibited solutions to obtain more information about the corrosion inhibition mechanism of the carbon steel (CS). EIS results were displayed as Nyquist plots in Figure 3. In our data, the semicircles of the EIS experiments were slightly depressed compared to those derived from the theory of EIS. The imperfectness of the capacitive loop is a typically observed behavior, and it is assigned as a result of heterogeneity, the roughness of the steel surface, and frequency dispersion [14,15]. Following 60 min of soaking the electrodes in the imperative concentration (open circuit potential), the plots were received.

**Figure 4:**
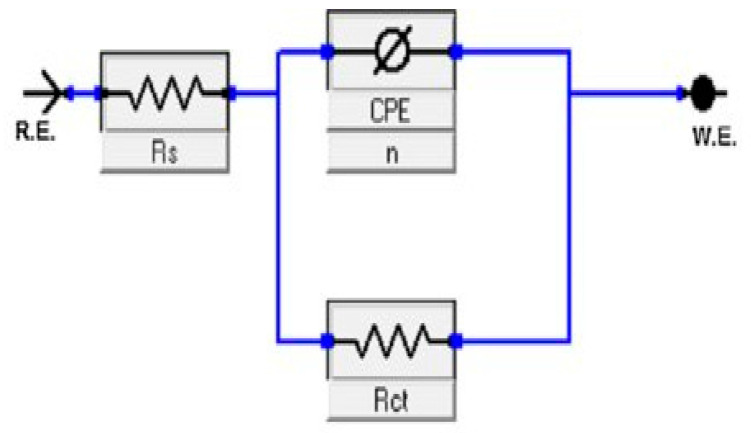
Equivalent circuit model for the analysis of the electrochemical impedance spectroscopy data.

It is clear from Figure 3 that single capacitive loops have been obtained for all inhibitors, indicating that the charge transfer process controlled the carbon steel (CS) dissolution at the metal/solution interface. The inhibition efficiencies, EI_Rct_ (%), are shown in Table 3. The Electrical Equivalent Circuit (EEC) formula usually used for adjustment is shown in Figure 4.

In order to determine R_ct_ values, the high-frequency impedance was subtracted from the low-frequency one, as shown in Equation (5):

R_ct_ = Z_re_ (at low frequency) − Z_re_ (at high frequency) (5)

C_dl_ values (electrochemical double layer) were ascertained at the frequency f_max_ when the imaginary part of the impedance had a supreme value (−Z_max_) by Equation (6) [10]:

(6) $$C_{\mathrm{dl}}=\frac{1}{2{\pi f}_{\max}R_{\mathrm{ct}}}$$

The inhibition efficiency IE%_(EIS)_ was calculated by using Equation (7):

(7) $${\mathrm{EI}\%}_{\left(\mathrm{EIS}\right)}=\frac{R_{\mathrm{ct}}^{o}-R_{\mathrm{ct}}}{R_{\mathrm{ct}}^{o}}\times 100$$

where $R_{\mathrm{ct}}^{o}$ and $R_{\mathrm{ct}}$ are the charge transfer impedance values without and with the appearance of the inhibitor, respectively.

Table 3 reveals that the resistance values increased in the presence of inhibitor. This can be ascribed to the influence of corrosion protection of the molecules. It was also found that C_dl_ values decreased in the appearance of the inhibitor, which may be assigned to the local dielectric constant decrease and the rise in the depth of the electric double layer [16,17]. This, in turn, indicates that the inhibitor molecules were working by adsorption at the solution/metal interface. The reduction of C_dl_ values, the increase of R_ct_ values, and (henceforward) the IE% increase is likely due to the ordinary substitution of water molecules by the adsorbed inhibitorq1 molecules on the steel surface, diminishing the degree of iron oxidation [18,19]. The outcomes achieved from the EIS tests are in excellent agreement with those collected from polarization tests.

### 2.5. Adsorption Study

The adsorption study intends to determine the way by which the inhibitor molecules interact with the steel surface. Values of surface coverage, θ, at multiple inhibitor concentrations at 298 K, exhibited in Table 1, have been manipulated to ascertain the adsorption isotherm. θ values were calculated by using Equation (8):

(8) $$\theta =\frac{W_{\mathrm{corr}}^{o}-{\;W}_{\mathrm{corr}}}{W_{\mathrm{corr}}^{o}}$$

There are various patterns of adsorption isotherms such as Freundluich, Temkin, and Langmuir isotherms.

**Figure 5:**
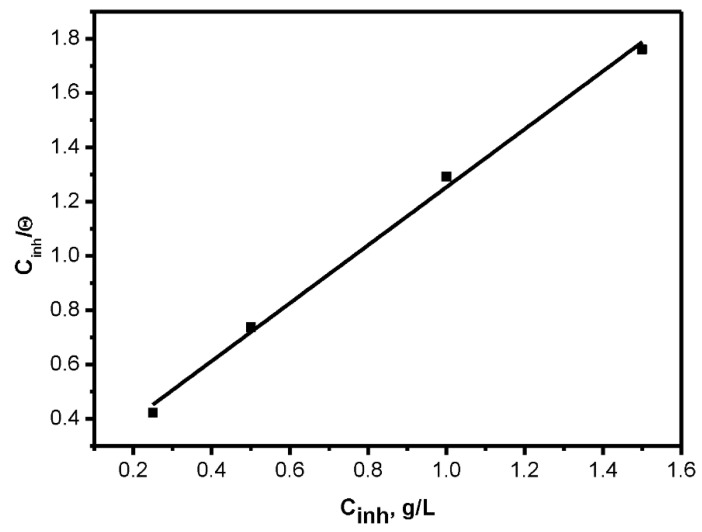
Langmuir isotherm plot of adsorbed on CS in sea water.

Application of Equation (9) gives straight lines with slope values of 1.068 and a good correlation coefficient (R^2^ = 0.997), proving that the adsorption of the inhibitor molecules from seawater on the steel surface follows the Langmuir model. Results are shown in Figure 5.

(9) $$\frac{C_{\mathrm{inh}}}{\theta }=\frac{1}{K_{\mathrm{ads}}}+C_{\mathrm{inh}}$$

(10) $$\Delta G_{\mathrm{ads}}=-\mathrm{RT}\;\ln\;\left(K_{\mathrm{ads}\;}\times 999\right)$$

Values of equilibrium adsorption constant (K_ads_) were measured from the intercept of Equation (16), and ΔG_ads_ values were calculated from Equation (10). Results are shown in Table 4.

Results in Table 4 show that values of adsorption free energy are negative, which implies that the adsorption process was spontaneous. According to results published earlier, it seems that when the value of $\Delta G_{\mathrm{ads}}$ ∼−40 kJ/mol (or more negative), the rule is considered to be chemisorption, whereas if $\Delta G_{\mathrm{ads}}$ ∼−20 kJ/mol (or less negative), the manner is feasibly figured to be physio-sorption. In this study, the results signify both physical and chemical interactions [20,21,22].

### 2.6. Effect of Temperature

**Figure 6:**
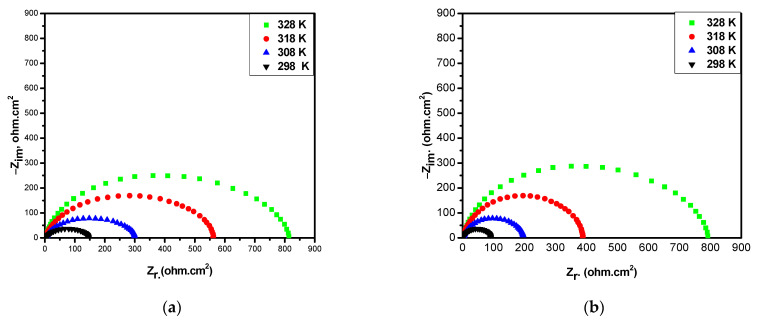
Nyquist plots for CS in sea water at different temperatures for (**a**) BTA and (**b**) Blank.

The EIS technique was modified in order to investigate the effect of temperature on the inhibition process and to gain some thermodynamic parameters of the corrosion means. EIS tests were conducted at a temperature range (298–328 K) with and without the optimal concentration of the inhibitors (Figure 6).

The results displayed in Table 5 confirm that the values of charge transfer resistance (R_t_) decreased as the temperature increased in both inhibited and uninhibited media. Table 5 also revealed that IE% values in the presence of all inhibitors slightly decreased as the temperature increased. This could be due to the fact that the desorption process may take place at elevated temperatures. These results prove that the BTA is an efficient inhibitor over the studied temperature range [22,23].

**Figure 7:**
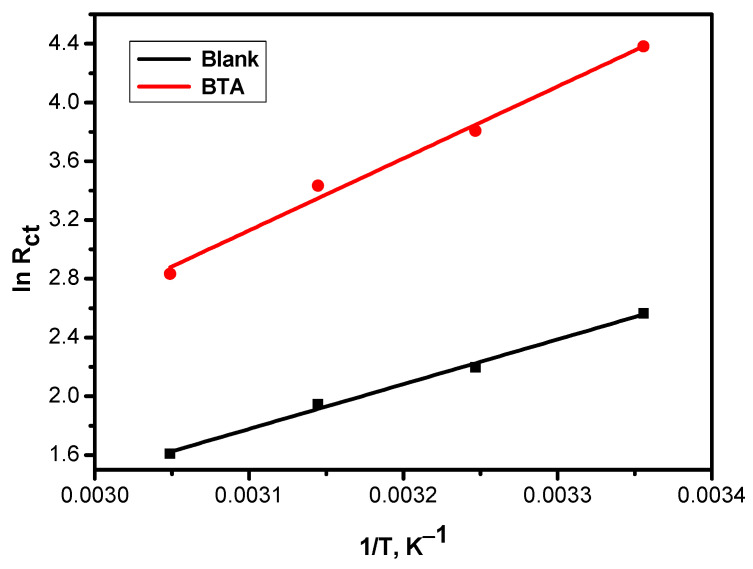
Arrhenius plots of CS in sea water with and without 1.50 g/L of various inhibitors.

**Figure 8:**
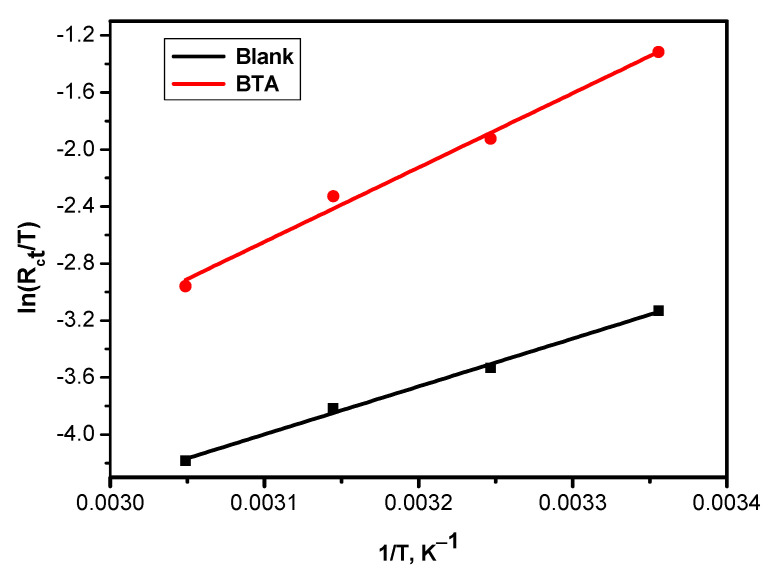
Erying plots of CS in sea water with and without 1.50 g/L of various inhibitors.

The charge transfer resistance (R_ct_) was applied to assess the activation energy values shown in the Arrhenius Equation (Equation (11)) and Figure 7. In contrast, the entropy change (∆S) and enthalpy change (∆H) were obtained from the intercept and slope of Erying equation (Equation (12)) and Figure 8, respectively.

(11) $$\ln R_{t}=\ln A-\frac{E_{a}}{\mathrm{RT}}$$

(12) $$\ln\frac{R_{t}}{T}=\frac{-\Delta H}{\mathrm{RT}}+\ln\frac{k_{B}}{h}+\frac{\Delta S}{R}$$

where E_a_ is the activation energy of the corrosion manner, T is the absolute temperature, k_B_ is the Boltzmann constant, A is the frequency factor, and h is Planck’s constant.

Values of E_a_, ΔS, and ΔH with and without 1.5 g/L are shown in Table 6. It is clear that the value of activation energy E_a_, (i.e., of the corrosion process in the absence of the BTA) was lesser than that in the presence of the BTA, confirming that the corrosion process became more difficult after adding the inhibitors [24].

The thermodynamic parameters (ΔH and ΔS) in the presence of the BTA were more extended than those calculated in the absence of the BTA. The further positive sign of ΔH in the presence of the inhibitor designates the endothermic nature of the corrosion process, suggesting that the ionization of the steel surface was slow [25] in the presence of the inhibitors. The significant and positive value of entropy (ΔS) denotes that during the rate-determining step, the activated complex was reached via an association rather than a dissociation step [26], confirming that an increment in disordering occurred when going from reactants to the activated complex [27].

### 2.7. Investigation Morphology of Steel Samples by Scanning Electron Microscopy (SEM)

**Figure 9:**
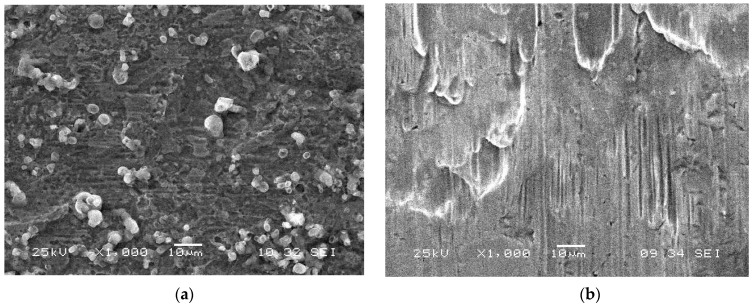
SEM photographs of CS dipped for 6 h in (**a**) seawater and (**b**) seawater with 1.5 g/L of the BTA at 298 K.

The steel samples were characterized before and after their immersion in seawater by SEM to provide visual data on how the inhibitors affected the morphology of the CS surface. The results are displayed in Figure 9. As the figure shows, the CS surface without inhibitor was highly corroded and damaged due to rapid corrosion attack in seawater. In comparison, significant improvements were observed in the presence of 1.5 g/L of inhibitor due to the involvement of more inhibitor constituents in the interaction with the reaction sites of the CS surface.

### 2.8. Comparison of the Efficiency of the Inhibitors with Other Inhibitors

Results displayed in Table 7 reveal that the BTA can be considered a potent inhibitor. It is clear that the efficiencies of most of the plant extracts used as corrosion inhibitors lie in the range 79–89%. The BTA can be considered as one of the top eco-friendly corrosion inhibitors.

### 2.9. DFT Calculation

**Figure 10:**
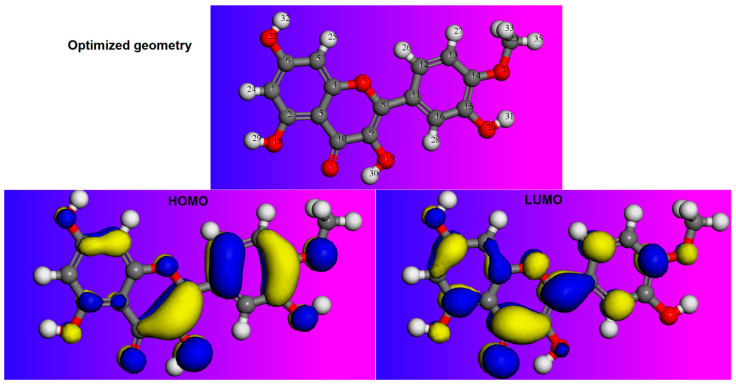
The optimised geometry, i.e., LUMO and HOMO orbitals of the OMPL molecule obtained using the DFT method.

The DFT methods in corrosion science are primarily based on theoretical concepts investigating the relationship between inhibitor structures and their inhibition property. It could be argued that Frontier orbital energies, chemical reactivity descriptors such as electron affinity, ionization energy, electronegativity, and the fraction of electrons transferred offer significant shreds of evidence about inhibition efficiency of inorganic and organic molecules against CS corrosion. In this work, DFT calculations were done in a liquid phase and optimized molecular structure; HOMO and LUMO of the studied compound are graphically presented in Figure 10. Looking at Figure 10, it was evident that the distribution of the electron density in the HOMO orbital covered the entire molecular structure of the BTA. These results are due to the presence of the conjugation effect and the high reactivity of the studied compound, which contains oxygen atoms and hydroxyl groups, and could have increased the electron-donating ability of the molecule.

Moreover, we note that the LUMO electron density is almost similar to the HOMO distribution. This observation is an indication of its ability to receive electrons from the steel surface. Therefore, the findings above revealed that metal-inhibitor interactions could have occurred via donation and back-donation of the electrons and electrostatic interactions.

Furthermore, some of the quantum chemical descriptors such as E_HOMO_, E_LUMO_, and ΔN_110_ are listed in Table 8. Consistent with the literature, it is well known that the energy of LUMO controls the electron-accepting aptitude of the molecule while the HOMO energy determines the prediction of the electron-donating ability [33,34,35]. Moreover, the ΔN value is an important index widely recognized to help predict inhibitor molecules’ chemical reactivity and stability. Generally, it seems that the strong interaction of an inhibitor molecule with a steel surface occurs at low values.

It can be noted that many plant extracts have been the subject of classic studies in the corrosion inhibition field. However, a search of the literature reveals few studies investigate the critical role of these inhibitor molecules based on the theoretical parameters obtained by the DFT method. Accordingly, it is not possible to discuss the energy gap of the inhibitor under study without comparing it with those of similar inhibitors. Nevertheless, the analysis of the DFT results represented in Table 8 showed the lower value of $\Delta E_{\mathrm{gap}}$ for the present molecule, suggesting that the BTA inhibitor has the highest capacity to hinder the CS corrosion. Furthermore, the calculated value of transferred electrons (ΔN_110_) is positive, indicating that the BTA inhibitor can transfer its electrons to the iron surface. This behavior confirms that the studied inhibitor has exceptional corrosion inhibition properties ascribed to its significant electronic properties.

### 2.10. MD Simulation

In this part of this study, molecular dynamic modelling set out to assess the adsorption of the BTA inhibitor on the surface of CS in a mimic condition. By using the MD approach, we have the chance to discover the adsorption profile that governs the interaction and bends energies between the BTA and the CS surface.

**Figure 11:**
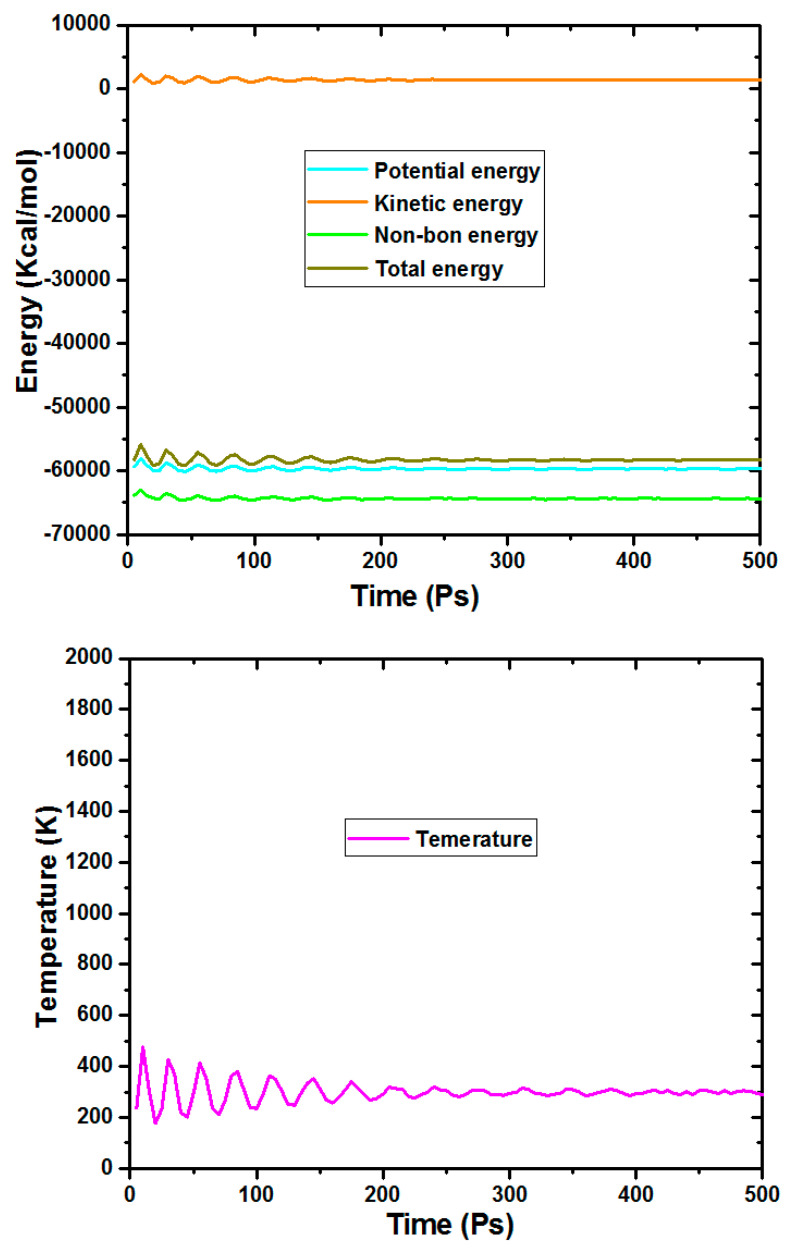
Temperature and energy equilibrium plots of the BTA adsorbed on the CS surface in solution.

**Figure 12:**
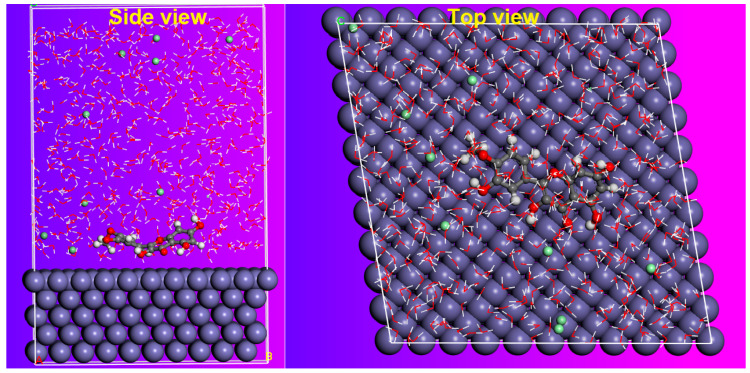
Final adsorption configurations of BTA on the most stable plane of Fe(110) at 298 K.

As described in the experimental section, MD simulations have been implemented in the liquid solution at NVT conditions, and the energy fluctuation and temperature plots have been displayed in Figure 11. From the chart, was evident that the equilibrium state of the BTA is kept constant. The last adsorption configuration of the BTA, adsorbed on the most stable iron (110) at 298 K with other corrosion particles, is shown in Figure 12. The most exciting aspect of this graph is that the BTA molecule is located near the iron surface, and is adsorbed with a planar arrangement that can help ensure maximum coverage of the surface area. Notably, the extent of interaction shown by a parallel configuration for the BTA is principally affected by the adsorption sites in the target molecule, leading to a high interaction with the metallic surface.

Without a doubt, oxygen atoms, hydroxyl groups, and phenyl rings positively enhance the interaction process between the inhibitor and steel surface. The adhesion strength between the BTA compound and the CS surface of binding energies and interaction was also evaluated. As mentioned in the literature review, higher interaction energy reflects the strong adsorption between an inhibitor compound and an iron surface [36]. This study found that the BTA inhibitor showed a strong interaction and bending affinity towards the iron surface, wherein the E_interaction_ value was −568.34 kJ mol^−1^ (E_bending_ = 568.34 kJ mol^−1^). According to these data, we can assume that the more significant negative value signifies that the adsorption of the BTA on the surface of the CS is permanent, stable, and spontaneous. Furthermore, the large amount of the binding energy suggests that the BTA was adsorbed with the aid of more than one reactive site. These findings are also in good agreement with experimental and DFT studies, which found that the inhibitor extracted from barks of *Tamarix aphylla* under study showed strong inhibition properties against CS.

## 3. Materials and Methods

### 3.1. Collection and Pretreatment of the Plant Sample

Barks of *T. aphylla* were collected from the areas around Khamis Mushait and Abha in the Aseer region, Saudi Arabia. The barks were rinsed carefully in tap water, followed by bi-distilled water, to get rid of soil particles and were cut into small pieces and air-dried under shade at room temperature for two weeks. The dried samples were later ground to a fine powder to produce 400 g of the bark of *T. aphylla*.

### 3.2. Extraction of Plant Samples

The plant sample was macerated in aqueous ethanol (ethanol: water, 80% *v*/*v*) at room temperature for five days with occasional stirring. The extract was collected every 36 h by decantation, and the fresh solvent was added to the residue. Collected extracts were filtered through Whatman No. 1 filter paper, combined and concentrated to dryness under reduced pressure at 313 K using a rotary evaporator (IKA R10, C S99-China). The obtained concentrated extracts were weighed and stored in a refrigerator (269 K) until used for analyses.

### 3.3. Scanning and Verification of Extract

#### 3.3.1. Phytochemical Screening of the Crude Extracts

The ethanolic extract of the plant bark was subjected to preliminary phytochemical screening to detect various phytoconstituents (alkaloids, saponins, glycosides, flavonoids, tannins, triterpenes, anthraquinones, and phenolic compounds) by adopting standard protocols [37].

#### 3.3.2. Phytochemical of *Tamarix aphylla* Barks

**Figure 13:**
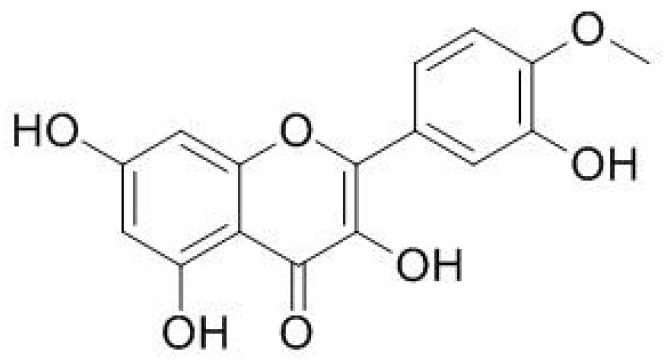
Chemical stucture of tamarixetin.

The gas chromatography–MS analysis exhibited that the barks of *T. aphylla* contain ten different compounds. Among these ten, the dominant compound is tamarixetin (Figure 13) [38,39].

Most types of equipment used in the desalination plants are made of CS, consisting of 1.8% C, 0.241% Si, 0.439% Mn, 0.190% Cu, and Fe balance. Steel samples were collected from the Alshqiq desalination plant, south of Saudi Arabia.

### 3.4. Corrosion Measurements

#### 3.4.1. Mass Loss Measurements

Dimensions of the samples used throughout all tests were 2.0 × 2.0 × 0.8 cm. For the surfaces of all samples, polishing was done using different SiC grit papers (1200, 800, 320 and 180 grades) which were then flushed thoroughly with bi-distilled water, degreased, and dehydrated with acetone. Mass loss measures were conducted at 298 K for 6, 12, 18, and 24 h. All measurements are based on the ASTM G1 standard.

#### 3.4.2. Electrochemical Tests

The electrochemical experiments were performed using a potentiostat Gamry interface 1000No—06094 (Gamry, Warminster, PA USA) led by Framework 7.07 software. A cell with three electrodes was attached to the thermostat. A Pt electrode and calomel electrode were utilized as auxiliary and referenced electrodes, respectively. Likewise, the material was applied for both gravimetric and electrochemical experiments. Potentiodynamic polarization (PP) tests were performed at a scan rate of 1.0 mV/s. Before all of the experiments, the potential was allowed to be stable at free potential during 1.0 h. The polarization curves were attained from −1000 mV to 1000 mV. Some experiments were employed at 298–328 K temperature range in order to investigate the effects of temperature on inhibitors effectiveness,

Measurements of electrochemical impedance spectroscopy (EIS) were performed using the related instrument (Gamry interface 1000). Prior to sine wave voltage (10 mV) peak to peak, wavelengths between 10^4^ Hz and 10^−3^ Hz were superimposed on the resting potential and the steady-state current at a corrosion potential was ascertained., Computer programs automatically controlled the measurements conducted at rest potentials after 1 h of contact at 298 K. The EIS plots were taken as Nyquist plots. All tests were performed three times to confirm reproducibility.

### 3.5. Scanning Electron Microscope (SEM) Studies

In order to investigate the morphology of the surface of the CS, samples were scanned using a Jeol 6360 (Japan) scanning electron microscope after 6 h of immersion in seawater with and without 1.5 g/L of the BTA (which underwent the same pretreatment previously described in the gravimetric experiments).

### 3.6. Theoretical Evaluation

#### 3.6.1. DFT Details

In this research, quantum chemical calculations were implemented to better understand the critical role of eco-friendly inhibitors extracted from the barks of *Tamarix aphylla* during the corrosion inhibition process. For that purpose, the geometry optimization of the studied inhibitor and the calculations of quantum chemical parameters were performed using ADF2020 packages [40] and DMol^3^, respectively. The geometry optimizations were first computed for the studied inhibitor. Then, the DMol^3^ was employed as a reliable module integrated with the high-performance software (Materials Studio version 6.0) [41]. All calculations were carried out using generalized gradient first principles approximation (GGA) and Perdew, Burke, and Ernzerhof formalism (known as PBE) with double numeric basis sets plus polarization (DNP) in the COSMO implicit solvent model [42,43]. At the end of DFT analysis, the molecular structure of the inhibitor under study was thoroughly analyzed, and the optimized structure was used to estimate some proper derived parameters. By these calculations, the frontier molecular orbitals, such as the energy of the highest occupied molecular orbital (E_HOMO_) and energy of the lowest unoccupied molecular orbital (E_LUMO_), were estimated by applying Koopmans’ theorem [44,45]. Furthermore, in the light of HOMO and LUMO energies, the values of energy gap (ΔE), electronegativity, and global hardness were calculated. By applying Koopmans’ theorem, one can write the following Equations [20]:

(13) $$\mathrm{IE}=-E_{\mathrm{HOMO}}$$

(14) $$\mathrm{EA}=-E_{\mathrm{LUMO}}$$

(15) $$\mathrm{Absolute}\;\mathrm{electronegativity}\;\chi =\frac{\mathrm{IE}+\mathrm{EA}}{2}$$

(16) $$\mathrm{Absolute}\;\mathrm{hardness}\;\eta =\frac{\mathrm{IE}-\mathrm{EA}}{2}$$

(17) $$\Delta E={\;E}_{\mathrm{LUMO}}-E_{\mathrm{HOMO}}$$

The Pearson method was used by applying the following Equation [46,47].

(18) $$\Delta N=\frac{\phi -{\;\chi }_{\mathrm{inh}}}{2\left(\eta_{\mathrm{Fe}}+{\;\eta }_{\mathrm{inh}}\right)}$$

where (ΔN) represents the fraction of transferred electrons, and IE is the ionization energy. The work function (ϕ) of the Fe(110) was generally known to be 4.82 eV, while the hardness of iron was taken as 0 since IE = EA for bulk metals [48,49].

#### 3.6.2. Molecular Dynamic Simulations Details

In recent years, researchers have investigated various approaches to give more insight into the complex adsorption phenomena of inhibitor molecules on metal surfaces during the corrosion inhibition process. For this purpose, one of the most well-known tools for assessing the adsorption behavior of inhibitor molecules on metal surfaces is molecular dynamic (MD) simulation. The functional properties of the BTA particles, in terms of intermolecular interactions between the BTA particles and CS surface, have been studied using the MD approach. In this work, MD simulations were performed using Forcite calculation, amorphous construction, and Build layer modules implemented in the Material Studio program. Firstly, the Fe(110) surface with a slab of 5 Å was chosen, as this iron surface is associated with high stabilization energy with a highly packed structure. Secondly, MD simulations were modelled in a simulation box (25.28 × 25.28 × 38.32 Å^3^) that covers the BTA inhibitor and corrosive molecules such as Cl^−^, H_2_O, and H_3_O^+^ ions. Furthermore, using an Anderson thermostat, MD simulations were achieved in the NVT canonical ensemble at temperatures of 298 K with a time-step of 0.1 fs and a simulations time of 500 ps. For MD modelling, the COMPASS force field (condensed phase optimized molecular potentials for atomistic simulation studies) was used for geometry optimization of the molecular structure., Some fruitful insights into the characteristics of steel-inhibitor interactions can also be gained from discussing the extent of adsorption of BTA based on interaction and binding energies obtained when the studied systems reach their equilibrium. The interaction and the binding energies ($E_{\mathrm{Binding}}=-E_{\mathrm{interaction}}$) can be estimated using the following equation [50,51]:

(19) $$E_{\mathrm{interaction}}={\;E}_{\mathrm{total}}-\left(E_{\mathrm{surface}+H_{2}O+{\;H}_{3}O^{+}+{\;\mathrm{Cl}}^{-}}+{\;E}_{\mathrm{inhibitor}}\right)$$

In the above equation, E_total_ represents total system energy,${\;E}_{\mathrm{surface}+H_{2}O+{\;H}_{3}O^{+}+{\;\mathrm{Cl}}^{-}}$ is the total energy of Fe(110) and corrosive solution without the adsorbed BTA particles, and Inhibitor represents the total energy of the BTA particles.

## 4. Conclusions

It can be concluded from the present work that the examined bark extract of *Tamarix aphylla* (BTA) is a good inhibitor for CS corrosion in sea water at the temperature range between 298 and 328 K. Mass loss investigations proved that the efficiency of the BTA reached 85.2%. The potetiodynamic polarization technique showed that the BTA suppresses both cathodic and anodic reactions. Thus, the BTA is classified as a dual-type inhibitor. The EIS investigations proved that the addition of the BTA to the corrosion medium increases the inhibition efficacy. It was also found that the adsorption of the BTA follows the Langmuir model. DFT calculations of the BTA exhibited that its adsorption over the CS surface principally depends upon electron donation. MD simulations confirmed a high adsorption affinity of BTA molecule to the CS surface.

## Figures and Tables

**Table 1:** Weight loss results of steel samples in the presence and absence of various concentrations of BTA at 298 K.

Inhibitor	C (g/L)	C.R (mm·y^−1^)	EI_WL_ %	θ
Blank	-	1.124	-	-
BTA	0.25	0.458	59.2	0.592
	0.50	0.362	67.7	0.677
	1.00	0.254	77.4	0.774
	1.50	0.166	85.2	0.852

**Table 2:** Electrochemical parameters of steel at numerous concentrations of the inhibitors in seawater and corresponding inhibition efficiency.

Inhibitor	C (g/L)	−E_corr_ (mV/SCE)	I_corr_ (μA cm^−2^)	b_a_ (mV dec^−1^)	−b_c_ (mV dec^−1^)	IE_Icorr_ (%)
Blank	-	467	1437	157	227	-
BTA	0.25	511	611	168	188	57.4
	0.50	498	452	172	199	68.5
	1.00	519	297	191	222	79.3
	1.50	503	219	181	201	84.8

**Table 3:** Electrochemical impedance parameters for corrosion of the iron specimen at variable concentrations of the inhibitors at 298 K.

Inhibitor	C (g/L)	R_ct_ (Ω·cm^2^)	Q × 10^−4^ s^n^/(Ω·cm^2^)	(10^4^) C_dl_(μF/cm^2^)	EI_Rct_ (%)
Blank	-	13	2.65	145.5	-
BTA	0.25	30	2.43	60.3	56.7
	0.50	33	2.23	41.4	60.6
	1.00	61	2.03	27.9	78.6
	1.50	79	1.87	8.9	83.5

**Table 4:** Values of $K_{\mathrm{ads}}$ and $\Delta G_{\mathrm{ads}}$.

Inhibitor	**K_ads_**	**ΔG_ads_** **(kJ/mol)**
BTA	5.38	−21.3

**Table 5:** Influence of temperature on the adsorption of different inhibitors (1.50 g/L) in seawater on the CS at different temperatures.

Inhibitor	T (K)	R_ct_ (Ω·cm^2^)	Q × 10^−4^ (s^n^·Ω^−1^·cm^−2^)	C_dl_ (µF/cm^2^)	EI_Rct_ (%)
Blank	298	13	2.65	145.5	---
	308	9	2.86	117	---
	318	7	2.98	130	---
	328	5	3.23	87	---
BTA	298	80	1.87	32	83.5
	308	45	2.03	48	80.0
	318	31	2.35	61	77.4
	328	17	2.59	83	70.7

**Table 6:** Values of activation parameters for CS in seawater in the absence and presence of 1.50 g/L of the BTA.

Inhibitor	E_a_ (kJ/mol)	∆H (kJ/mol)	∆S (J/mol)
Blank	25.3	27.9	317.2
BTA	41.1	43.4	355.5

**Table 7:** Comparison of maximum inhibition effect (IE%) for different inhibitors.

Plant Type	Maximum IE%
*Coleus forskohlii* Leaf Extract [28]	87.5
*Psidium Guajava* Seeds [29]	81.2
*Khayasenegalensis* Leaves [30]	89.0
*Rotula aquatic* Leaf Extract [31]	79.6
*Capparisspinosa* Leaf Extract [32]	79.3
BTA This Study	85.2

**Table 8:** The quantum chemical parameters for BTA analyzed from DFT method in aqueous solution.

Molecule	E_HOMO_ (eV)	E_LUMO_ (eV)	∆E_gap_ (eV)	EA (eV)	IE (eV)	χ (eV)	∆N_110_
BTA	−5.117	−2.137	2.980	5.117	2.137	3.627	0.400

## Data Availability

Data are available from the authors upon request.
